# Sensitisation patterns to tomato seed

**DOI:** 10.1186/2045-7022-5-S3-P120

**Published:** 2015-03-30

**Authors:** Miguel González, Laura Martín-Pedraza, Maria Luisa Somoza, Natalia Blanca-López, Maria Luisa Macías, Diana Perez, Mayte Villalba, Cristobalina Mayorga, Gabriela Canto, Maria Jose Torres, Ana Aranda, Ana Molina, Miguel Blanca

**Affiliations:** 1 Research Laboratory, IBIMA, Regional University Hospital of Malaga, UMA, Malaga, Spain; 2 Biochemistry and Molecular Biology Department, University Complutense Madrid, Spain; 3 Allergy Service, Infanta Leonor Hospital, Madrid, Spain; 4 Allergy Unit, IBIMA, Regional University Hospital of Malaga, UMA, Malaga, Spain

## Rationale

Food allergy is an increasing health problem with many proteins involved that belong mainly to a limited number of families. They show a high level of cross-reactivity. In the Mediterranean area, the most prevalent food allergens are those of vegetal origin. Although tomato (*Solanum lycopersium L.*) is one of the implicated foods, studies on the identification and relevance of their allergens have not been carried out in detail. This could be particularly relevant for tomato seeds as happen in other fruits like kiwi.

The aimed was to analyse the sensitisation pattern to tomato seeds in patients from two hospitals integrated in the RIRAAF.

## Methods

A large group of tomato-sensitized patients (N=96) was recruited. We included patients who suffered at least two episodes with tomato and/or having a positive skin prick test (SPT). Raw tomato seed extract was prepared and the protein profile characterized by SDS-PAGE. Patient sera were used for determining recognition profiles by western blotting.

## Results

Data from western blotting showed different patterns of IgE recognition. From all the bands those approx. of 10 kDa was the most frequently recognised in 46% of the patients. This band specifically appeared in 100% of serum from patients with anaphylaxis, 83% with urticaria, 0% with angioedema and 9% with OAS.

## Conclusions

These preliminary results show that a new seed protein from tomato could be a relevant allergen. Whether this is predictive of systemic reactions is being evaluated.

